# Risk-stratification in normotensive acute pulmonary embolism

**DOI:** 10.1007/s12471-014-0639-4

**Published:** 2014-12-11

**Authors:** M. L. Handoko, F. S. de Man

**Affiliations:** 1 Department of Cardiology and Physiology, VU University Medical Center / Institute for Cardiovascular Research, De Boelelaan 1117 - 5F013, 1081 HV Amsterdam, the Netherlands; 2 Department of Pulmonology and Physiology, VU University Medical Center / Institute for Cardiovascular Research, De Boelelaan 1117 - 4F002, 1081 HV Amsterdam, the Netherlands

In the Netherlands, 10,000 to 12,500 patients per year are diagnosed with acute pulmonary embolism (PE) [1]. The large majority of these patients are normotensive.

**Fig. 1:**
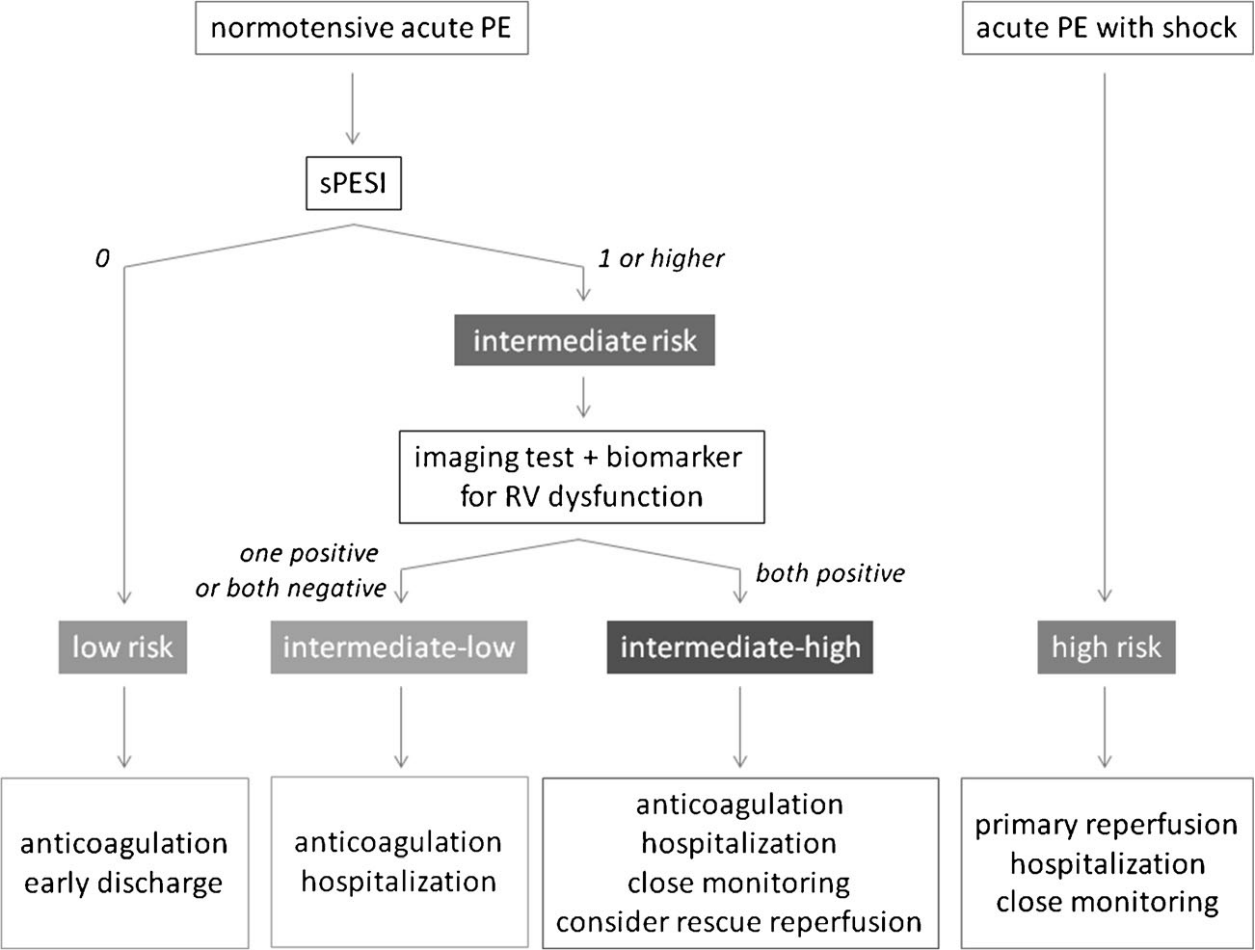
ESC risk-adjusted management strategies in acute pulmonary embolism, adopted from Konstantinides et al.[2] PE, pulmonary embolism; sPESI, simplified pulmonary embolism severity index; RV, right ventricular

Just recently, the European Society of Cardiology (ESC) published an update of their guideline on the diagnosis and management of acute PE [2]. Compared with the previous version of 2008, special emphasis was given to risk stratification in normotensive acute PE. The algorithm was largely based on the recent PEITHO trial [3]. In summary, the following prognostic assessment has been proposed (Fig. 1).

After establishment of the diagnosis of pulmonary embolism by CT angiography, a clinical assessment is performed by the simplified pulmonary embolism severity index (sPESI) [4]. To calculate sPESI, one point is scored for each of the following items: age > 80 years; history of cancer; history of heart failure and/or chronic lung disease; heart rate ≥110 bpm; systolic blood pressure < 100 mmHg; arterial oxyhaemoglobin saturation < 90 %. If sPESI is 0, the patient is considered at low risk (30-day mortality risk: 1.1 %), and is probably eligible for home treatment with either a vitamin K antagonist (plus low-molecular-weight heparin) or a new oral anticoagulant. If sPESI is 1 or higher, the patient is considered at intermediate risk (30-day mortality risk: 8.9 %) and additional testing is recommended, which includes an imaging test to evaluate signs of right ventricular (RV) dysfunction (either echocardiography or CT) and cardiac laboratory biomarkers (preferably troponin). When both tests return positive, the patient is classified as intermediate-high risk; if only one or neither test is positive, the patient is classified as intermediate-low risk. The guideline recommends initiation of anticoagulation and hospitalisation, and for intermediate-high risk patients even ‘close monitoring’ is advised. At the first sign of haemodynamic decompensation, rescue thrombolytic therapy is indicated.

RV failure due to pressure overload is considered the primary cause of death in severe PE. Therefore, it is very logical that the ESC guideline has incorporated imaging techniques that directly evaluate RV dysfunction. However, in practice this approach could be troublesome. First of all, even in the Netherlands, echocardiography and (to a lesser extent) CT angiography are not always available, especially during non-office hours. Secondly, the definition of RV dysfunction is poorly defined: given the peculiar geometry of the right ventricle, there is no individual (echocardiographic) parameter that provides fast and reliable information on RV size or function.

An alternative -and potentially more practical- approach could be the sole reliance on biomarkers of RV dysfunction. Increase of RV wall stress results in release of troponin and (NT-pro)BNP, and therefore increased plasma levels are found in intermediate to high risk patients. In this issue, Keller and coworkers provide us some insights into the relationship between cardiac troponin I and RV dysfunction [5]. Based on their retrospective analysis, they found a strong association between the two, consistent with what has been reported by others [6]. However, the positive and especially the negative predictive value of troponin I should be considered too low to solely guide clinical management (84 and 73 %, respectively). Meta-analysis by Becattini et al. showed that the prognostic performance of troponin T is similar to troponin I,[7] so no significant improvement can be expected from troponin T either.

So, what about (NT-pro)BNP? Recently Jiménez et al. prospectively evaluated the prognostic performance of BNP, along with sPESI, RV dysfunction detected by CT angiography or echocardiography, troponin I, and lower limb ultrasound testing (PROTECT study) [8]. They found that the combination of sPESI (of 0) and BNP (<100 pg/mL) gives a nearly perfect negative predictive value of 99 % (95 % confidence interval: 96–100 %) for a 30-day complicated course (death from any cause, haemodynamic collapse or recurrent PE). The positive predictive value was relatively low (26 %, 95 % confidence interval: 10–41 %). They concluded that this combination seems an excellent screening tool to identify low-risk PE patients from patients with intermediate risk. Interestingly, they did not find any support for the evaluation of RV dysfunction by either CT angiography or echocardiography as a prognosticator in normotensive PE, which was explained by the lack of a clear definition of RV dysfunction. The latter would imply that current ESC risk stratification strategy can be simplified even further.

In conclusion, the updated ESC guideline provides clear recommendations on risk stratification in PE. However, risk stratification in patients with normotensive PE remains suboptimal. Especially the subdivision of intermediate-low vs. intermediate-high PE patients might be unnecessarily complicated. Future studies that address this problem are needed.
